# Monitoring the patterns of submission and presence of tick-borne pathogens in *Ixodes scapularis* collected from humans and companion animals in Ontario, Canada (2011–2017)

**DOI:** 10.1186/s13071-021-04750-1

**Published:** 2021-05-17

**Authors:** Mark P. Nelder, Curtis B. Russell, Antonia Dibernardo, Katie M. Clow, Steven Johnson, Kirby Cronin, Samir N. Patel, L. Robbin Lindsay

**Affiliations:** 1 Enteric, Zoonotic and Vector-Borne Diseases, Health Protection, Operations and Response, Public Health Ontario, Toronto, ON Canada; 2 Field Studies, Zoonotic Diseases and Special Pathogens, National Microbiology Laboratory, Public Health Agency of Canada, Winnipeg, MB Canada; 3 Ontario Veterinary College, University of Guelph, Guelph, ON Canada; 4 Informatics, Knowledge Services, Public Health Ontario, Toronto, ON Canada; 5 Laboratory Surveillance and Data Management, Public Health Ontario, Toronto, ON Canada; 6 National Microbiology Laboratory, Public Health Agency of Canada, Winnipeg, MB Canada; 7 Bacteriology, Public Health Ontario, Toronto, ON Canada; 8 Department of Laboratory Medicine and Pathobiology, University of Toronto, Toronto, ON Canada

**Keywords:** *Anaplasma*, *Babesia*, *Borrelia*, One Health, Surveillance, Veterinary health, Zoonotic

## Abstract

**Background:**

The universal nature of the human–companion animal relationship and their shared ticks and tick-borne pathogens offers an opportunity for improving public and veterinary health surveillance. With this in mind, we describe the spatiotemporal trends for blacklegged tick (*Ixodes scapularis*) submissions from humans and companion animals in Ontario, along with pathogen prevalence.

**Methods:**

We tested tick samples submitted through passive surveillance (2011–2017) from humans and companion animals for *Borrelia burgdorferi*, *Borrelia miyamotoi*, *Anaplasma phagocytophilum* and *Babesia microti*. We describe pathogen prevalence in ticks from humans and from companion animals and constructed univariable Poisson and negative binomial regression models to explore the spatiotemporal relationship between the rates of tick submissions by host type.

**Results:**

During the study, there were 17,230 blacklegged tick samples submitted from humans and 4375 from companion animals. Tick submission rates from companion animals were higher than expected in several public health units (PHUs) lacking established tick populations, potentially indicating newly emerging populations. Pathogen prevalence in ticks was higher in PHUs where established blacklegged tick populations exist. *Borrelia burgdorferi* prevalence was higher in ticks collected from humans (maximum likelihood estimate, MLE = 17.5%; 95% confidence interval, CI 16.97–18.09%) than from companion animals (9.9%, 95% CI 9.15–10.78%). There was no difference in pathogen prevalence in ticks by host type for the remaining pathogens, which were found in less than 1% of tested ticks. The most common co-infection *B. burgdorferi* + *B. miyamotoi* occurred in 0.11% of blacklegged ticks from humans and animals combined. *Borrelia burgdorferi* prevalence was higher in unengorged (21.9%, 95% CI 21.12–22.65%) than engorged ticks (10.0%, 95% CI 9.45–10.56%). There were no consistent and significant spatiotemporal relationships detected via regression models between the annual rates of submission of each host type.

**Conclusions:**

While *B. burgdorferi* has been present in blacklegged ticks in Ontario for several decades, other tick-borne pathogens are also present at low prevalence. Blacklegged tick and pathogen surveillance data can be used to monitor risk in human and companion animal populations, and efforts are under consideration to unite surveillance efforts for the different target populations.

**Graphic Abstract:**

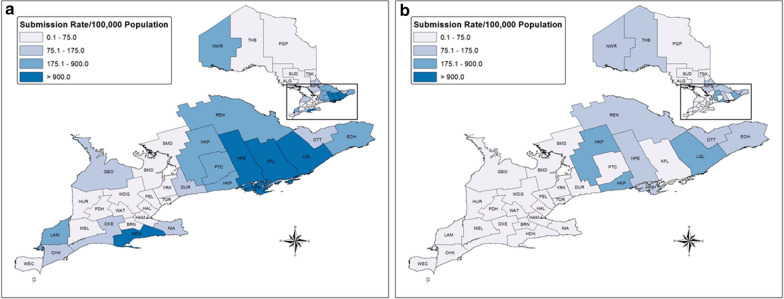

**Supplementary Information:**

The online version contains supplementary material available at 10.1186/s13071-021-04750-1.

## Background

Blacklegged ticks, *Ixodes scapularis*, transmit pathogens to humans and companion animals, particularly *Borrelia burgdorferi* sensu stricto, the causative agent of Lyme disease. Lyme disease is the most common vector-borne disease of humans in North America, with over 30,000 annual cases in the United States (USA) (2008–2016) [1]. In Canada, Lyme disease case reports continue to rise and, in 2017, approximately 1000 cases were reported in Ontario [2, 3]. Lyme disease is underreported in the USA, and researchers estimate there are more than 300,000 cases every year; however, the extent of underreporting in Canada is likely considerably less [4–6]. In 2015, 2799 (1.9%) of 146,046 dogs tested were seropositive for *B. burgdorferi* in Ontario and, while assumed to be low based on this percent seropositivity, the burden of disease in the province’s dogs is unknown [7, 8]. Blacklegged ticks transmit additional pathogens such as *Anaplasma phagocytophilum*, *Borrelia mayonii*, *Borrelia miyamotoi*, *Babesia microti*, *Babesia odocoilei*, Powassan virus (deer tick virus lineage) and *Ehrlichia muris eauclairensis* [9]. As the distribution of blacklegged ticks continues to expand in North America, aided by climate change and land use change, so does the risk of tick-borne diseases in humans and companion animals [2, 3, 10].

Blacklegged ticks from companion animals provide spatiotemporal data on Lyme disease risk to humans [11, 12]. In 2018, there were an estimated 8.3 million cats and 8.2 million dogs in Canada; 38% of Canadian households were estimated to have a cat and 41% to have a dog [13]. Worldwide, cats, dogs and humans have shared exposures to ticks and tick-borne pathogens for over 10,000 years [14]. In addition to Lyme disease, dogs are indicators of vector-borne disease risks in humans, such as Rocky Mountain spotted fever (*Rickettsia rickettsii*) and Chagas disease (*Trypanosoma cruzi*) [11, 15, 16]. Cats are less studied as indicators of vector-borne disease risks in humans; however, the body of work is increasing (e.g., detection of *Rickettsia* spp. in Spain) [17]. The ubiquity of the human–companion animal relationship provides a source of data for monitoring tick-borne diseases and, through a One Health approach, offers an opportunity for improving public and veterinary health.

In Ontario, tick-borne disease surveillance is undertaken through the monitoring and analysis of human reportable disease information [18, 19]. Tick populations and their pathogens are monitored through tick submissions by the public or healthcare professionals for identification and pathogen testing [2, 10, 20]. Passive surveillance (human and tick) provides public health officials the data needed to determine when and where to perform targeted active tick surveillance (i.e., tick dragging) which informs the identification of Lyme disease risk areas [21]. For companion animals, tick-borne disease surveillance is through several means, such as sentinel veterinary clinics and point-of-care serology [22, 23]. Monitoring ticks from companion animals is through the monitoring of web-based tick identification applications (e.g., Pet Tick Tracker, eTick) [24, 25]. Public and veterinary health is increasingly using each other’s data to assess disease risk. To assess the public and veterinary health threats posed by blacklegged ticks and their associated pathogens in Ontario, we describe the spatiotemporal trends for blacklegged tick submissions from humans and companion animals, as well as the pathogens identified from ticks (2011–2017, with some variability by pathogen). We tested the hypothesis that blacklegged tick submission rates from companion animals were temporally associated with blacklegged tick submission rates from humans within a geographic area (public health unit, PHU), potentially providing an early indicator of blacklegged tick populations in an area.

## Methods

### Study location

Ontario is the most populous province (14.2 million in 2017) in Canada and occupies approximately 100 million hectares, of which 52% is forested [26, 27]. Ontario's population is concentrated in the southern portion of the province (ca. south of 46° N), which is also where most blacklegged tick populations occur [10, 28]. This region is dominated by a moderate, humid, continental climate with a mixture of agricultural, suburban and urban landscapes. Oaks (*Quercus* spp.), maples (*Acer* spp.), yellow birch (*Betula alleghaniensis*), ash (*Fraxinus* spp.), basswood (*Tilia americana*), pines (*Pinus* spp.) and white spruce (*Picea glauca*) dominate the mixed forests of southern Ontario.

Thirty-six public health units (PHU) administered aspects of Ontario’s tick surveillance programs during the study; however, we based our analyses on the updated PHU geography that includes 35 PHUs. PHUs are further organized into seven health regions: Central East (DUR, HKP, PEL, PTC, SMD, YRK), Central West (BRN, HAL, HAM, HDN, NIA, WAT, WDG), Eastern (EOH, HPE, KFL, LGL, OTT, REN), North East (ALG, NPS, PQP, SUD, TSK), North West (NWR, THB), South West (CHK, GBO, HUR, LAM, MSL, OXE, PDH, WEC) and Toronto (TOR).

### Passive tick surveillance—human

We have described Ontario’s passive tick surveillance previously [9, 29]. Briefly, PHO identifies ticks found on humans submitted by the public through healthcare providers or PHUs and then sends blacklegged ticks to the National Microbiology Laboratory (NML) for pathogen detection. Data captured by the passive tick surveillance program included the submitter’s location of tick acquisition (city), city of residence, travel history, date of tick submission, and tick life stage and sex. If the location of tick acquisition was not specified, we used the city of residence to aggregate data at the PHU level; we expected the most likely exposure location was near or in the submitter’s city of residence [30]. Ticks in this report were either unengorged (no evidence of a blood meal) or engorged (slightly, partially or fully engorged). In 2014, the PHUs EOH, KFL and LGL ceased accepting tick submissions at their offices; however, healthcare providers could still submit ticks from patients.

### Passive tick surveillance—companion animals

In 2009, Public Health Ontario (PHO) ceased accepting ticks for identification and pathogen testing from non-human sources. Since 2010, veterinarians and citizens have submitted ticks from companion animals directly to the NML (Public Health Agency of Canada, PHAC) for identification and blacklegged ticks tested for pathogens. Ticks submitted from companion animals (i.e., cats and dogs) contained the same data fields as the human-derived ticks.

### Detection of tick-associated pathogens

For the purposes of analyses, unless otherwise specified, we will be reporting on submission-level results only (submissions may contain more than one tick). NML routinely tests blacklegged ticks submitted through passive tick surveillance for *B. burgdorferi*, *B. miyamotoi*, *A. phagocytophilum* and *Ba. microti* by real-time polymerase chain reaction (RT-PCR), as previously described [31, 32]. Briefly, we used QIAGEN DNeasy 96 tissue kits (QIAGEN Inc., Mississauga, Ontario) according to manufacturer’s protocol for DNA extraction. We used a duplex RT-PCR assay to screen the samples for *Borrelia* spp. and *A. phagocytophilum* by targeting the *23S rRNA* and *msp2* genes, respectively [33]. Analysis for *Ba. microti* was conducted using the methods described by Nakajima et al. (2009) for the detection of the *CCT eta* gene, followed by an in-house RT-PCR assay targeting the *18S rRNA* gene on positive samples [34]. We subsequently tested all *Borrelia* spp.-positive samples collected in 2014 for *B. burgdorferi* using a confirmatory *ospA* RT-PCR assay, and for *B. miyamotoi* using an *IGS* real-time PCR assay. We further verified *B. miyamotoi *positivity using the *glpQ* RT-PCR assay [31]. During 2014 and onwards, we confirmed *Borrelia* spp.-positive samples by *ospA* and *glpQ* assays only. We tested samples for *A. phagocytophilum* and *B. burgdorferi* from 2011 to 2017, *B. miyamotoi* from 2014 to 2017 and *Ba. microti* from 2013 to 2017.

We monitored each round of DNA extractions for cross-contamination by including at least two samples consisting only of nuclease-free water. Specific synthetic double-stranded DNA controls (Integrated DNA Technologies, Skokie, IL, USA) for each pathogen were included in each PCR run, while no-template controls consisting of master mix only served as negative amplification controls.

### Mapping and statistical analyses

We calculated PHU and provincial submission rates per 100,000 population for blacklegged tick submissions using population data from Statistics Canada via IntelliHEALTH Ontario (October 19, 2017). Ideally, we would use companion animal population numbers to calculate submission rates; however, accurate data on cat and dog populations are not available. Therefore, we use human population data as a proxy and assume companion animal ownership does not vary significantly over time and by geography within Ontario.

We used Excel v15.0 (Microsoft, Redmond, WA, USA; 2013) for descriptive statistics such as chi-square tests to determine independence of variables (e.g., percentage of ticks engorged by host) and *t* tests for difference among means (e.g., mean ticks per sample by host). We calculated maximum likelihood estimates (MLE) for pathogen prevalence in blacklegged ticks, with 95% confidence intervals (CIs), using the PooledInfRate v4.0 Excel add-in [35]. For co-infections, we removed all samples with multiple ticks from the dataset, as we could not confirm true co-infections in these samples (there may have been separate infections in separate ticks within the sample, and so we could not confirm that there was true co-infection). For co-infections, we calculated prevalence (no. positive samples/total single-tick samples tested). We created maps using Esri ArcGIS v10.3 (Esri, Redlands, CA, USA; 2014), using manual classification methods to classify PHU rates into categories.

A summary dataset was prepared that included the annual number of tick submissions from humans and animals for each PHU. The corresponding annual human populations in that PHU were included and annual summary rates were calculated in each PHU. Data were explored visually by plotting the annual rates of tick submissions from animals and humans by PHU. We constructed univariable Poisson regression models to explore the relationship between the rates of submission from humans and animals by PHU over time. For the first model, the dependent variable was the total number of tick submissions from humans in 2011 by PHU, with the offset of human population in each PHU in 2011. The independent variable was the total number of tick submissions from animals in each PHU in 2011. This analysis was repeated on data from 2012 to 2017. For each additional year, the total number of tick submissions from animals in the same year and all previous years were explored as independent variables to determine potential temporal influences. We assessed overdispersion for each univariable Poisson regression model. If overdispersion was evident (*α* < 0.05), then we used a negative binomial regression model. We conducted all regression modelling using STATA version 14.2 (StataCorp, College Station, TX, USA; 2018) and used a significance level of *α* = 0.05 for analyses.

## Results

### Blacklegged tick submissions from humans

**Fig. 1:**
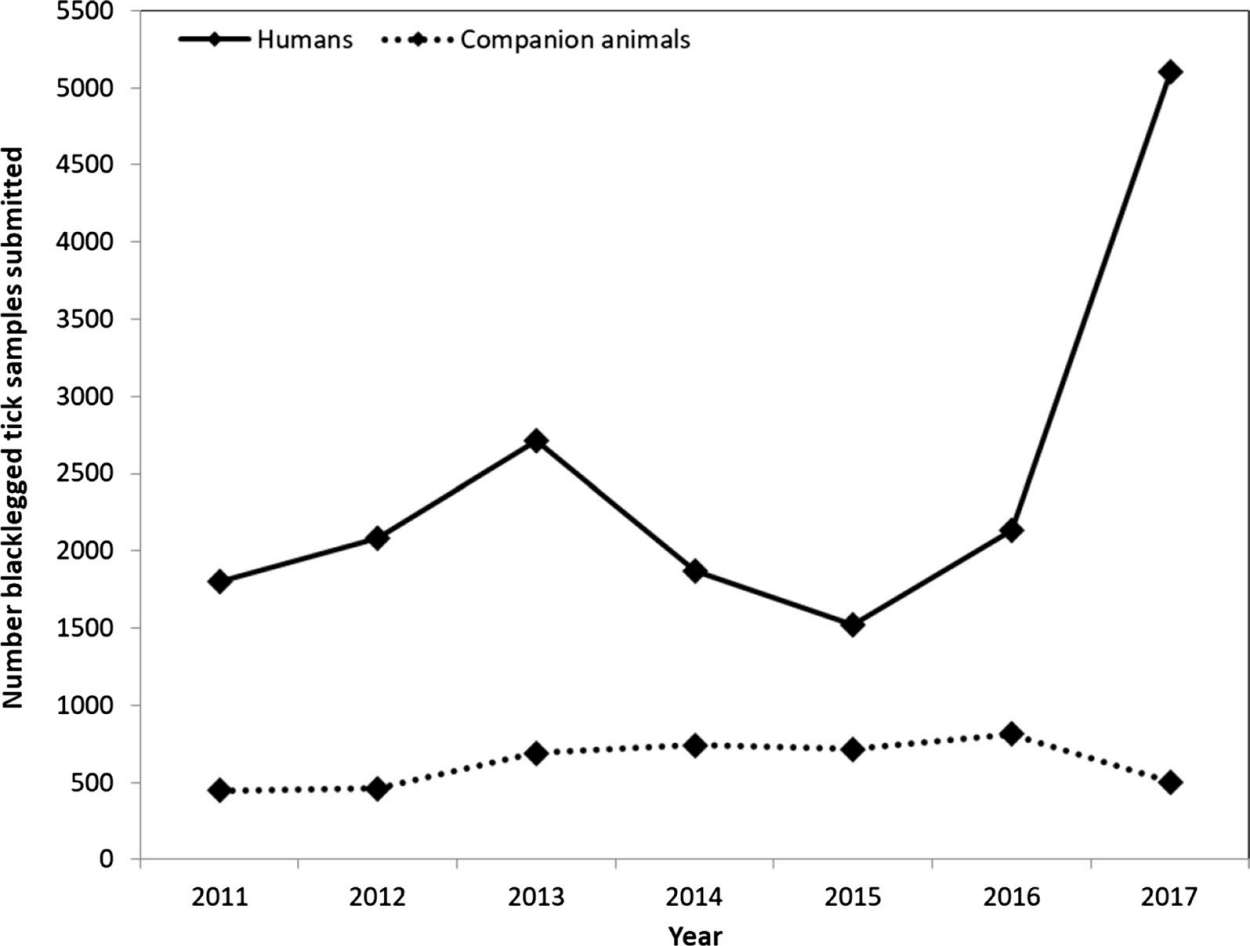
Number of blacklegged tick samples submitted from humans and companion animals: Ontario, Canada (2011–2017)

**Fig. 2:**
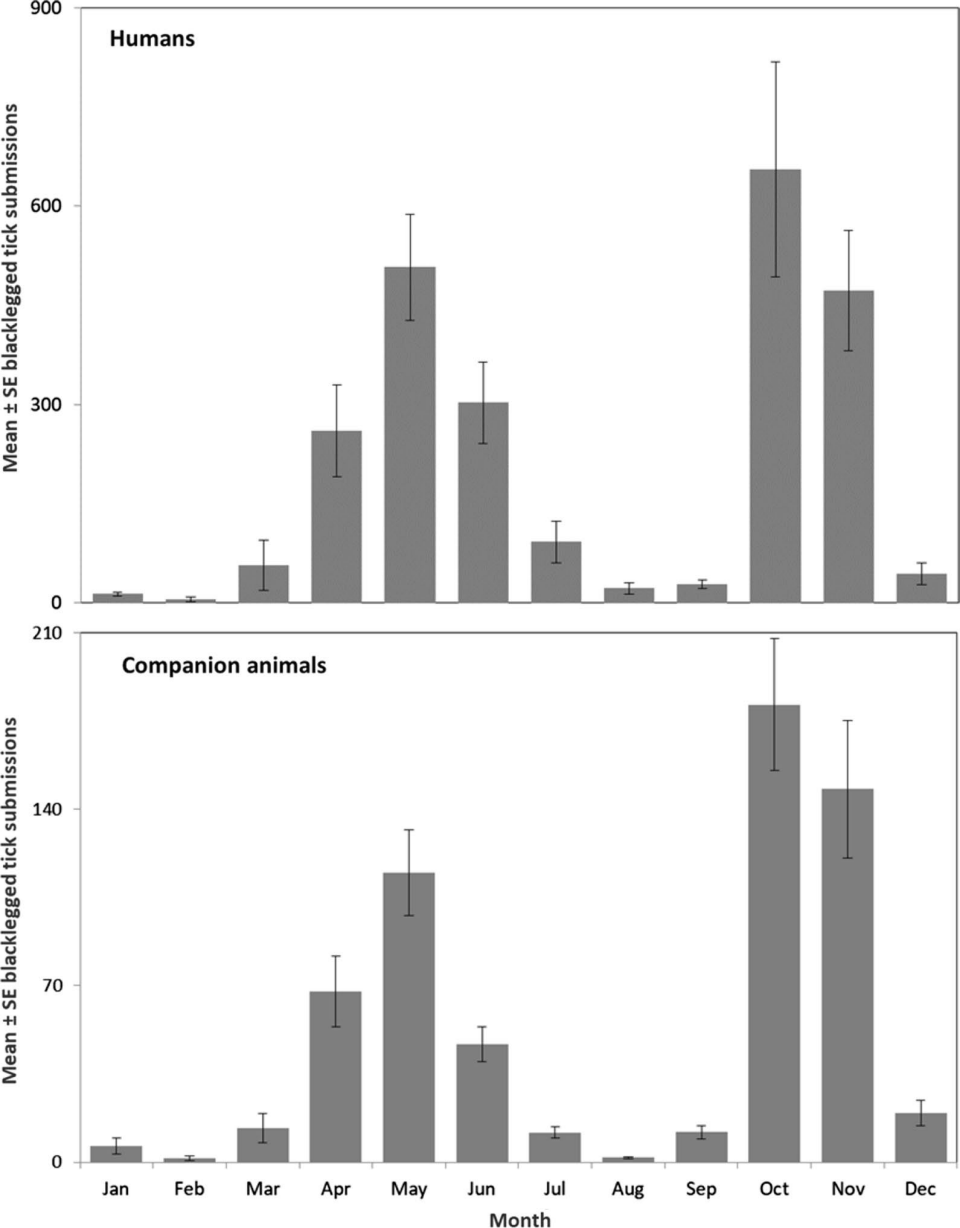
Mean blacklegged tick (all stages) samples submitted per month from humans and companion animals: Ontario, Canada (2011–2017)

**Fig. 3:**
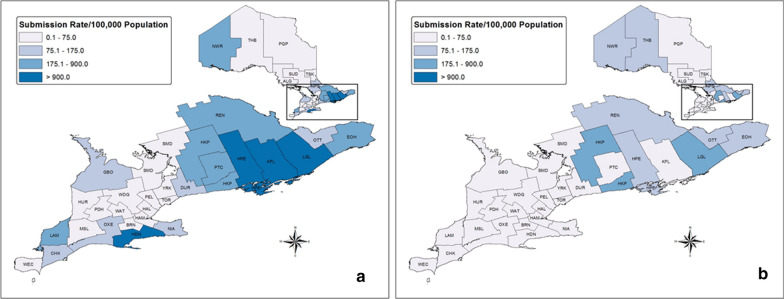
Blacklegged tick sample submission rates per 100,000 population: Ontario, Canada (2011–2017). *a humans, **b** companion animals. *ALG Algoma District, *BRN* Brant County, *CHK* Chatham-Kent, *DUR* Durham Regional, *EOH* Eastern Ontario, *GBO* Grey Bruce, *HAL* Halton Regional, *HAM* City of Hamilton, *HDN* Haldimand-Norfolk, *HKP* Haliburton–Kawartha–Pine Ridge District, *HPE* Hastings and Prince Edward Counties, *HUR* Huron County, *KFL* Kingston-Frontenac and Lennox & Addington, *LAM* Lambton, *LGL* Leeds-Grenville and Lanark District, *MSL* Middlesex-London, *NIA* Niagara Regional, *NPS* North Bay Parry Sound District, *NWR* Northwestern, *OTT* City of Ottawa, *OXE* Oxford Elgin-St. Thomas, *PDH* Perth District, *PEL* Peel Regional, *PQP* Porcupine, *PTC* Peterborough County-City, *REN* Renfrew County and District, *SMD* Simcoe Muskoka District, *SUD* Sudbury and District, *THB* Thunder Bay District, *TOR* City of Toronto, *TSK* Timiskaming, *WAT* Waterloo, *WDG* Wellington-Dufferin-Guelph, *WEC* Windsor-Essex County, *YRK* York Regional

We tested 17,230 samples of blacklegged ticks submitted from humans for pathogens of interest (2011–2017). The majority of ticks were adult females (91.0%) and unengorged (62.0%) (Table 1). Total tick samples per year varied from 1520 to 5099 (mean = 2669 ± 638.0), with an increase in submissions during the study period (Fig. 1). Most tick samples were submitted from April to June (43.5%) and October to November (45.8%) (Fig. 2). The highest rates of tick submissions were from LGL, HDN, KFL, HPE, HKP, EOH, REN, PTC, LAM and NWR (> 175 per 100,000 population) (Additional file 1: Table S1, Fig. 3).

**Table 1:** Summary of blacklegged ticks submitted from humans and companion animals: Ontario, Canada (2011–2017)

Blacklegged tick submission variable	Human	Companion animal
Total samples	17,230	4375
Total ticks	17,853	5438
Ticks per sample, mean ± SE (range)	1.0 ± 0.0021 (1–10)	1.2 ± 0.022 (1–58)
Tick submissions per year, mean ± SE (range)	2669 ± 638 (1520–5099)	625 ± 56.5 (447–812)
Tick samples, stage (%)*		
Female adult	91.0	96.0
Male adult	1.9	2.7
Nymph	6.8	0.5
Larva	0.2	< 0.1
Mixed stages	0.1	0.7
Tick samples, engorgement (%)**		
Engorged	37.7	92.0
Unengorged	62.0	6.0
Mixed engorgement	0.3	2.0

*SE* standard error

*Percentages based on: human, *n* = 17,228 and companion animal, *n* = 4371. Mixed stages, samples that include multiple life stages

**Percentages based on: human, *n* = 17,223 and companion animal, *n* = 4369. Mixed engorgement, samples that include ticks of different engorgement status

**Fig. 4:**
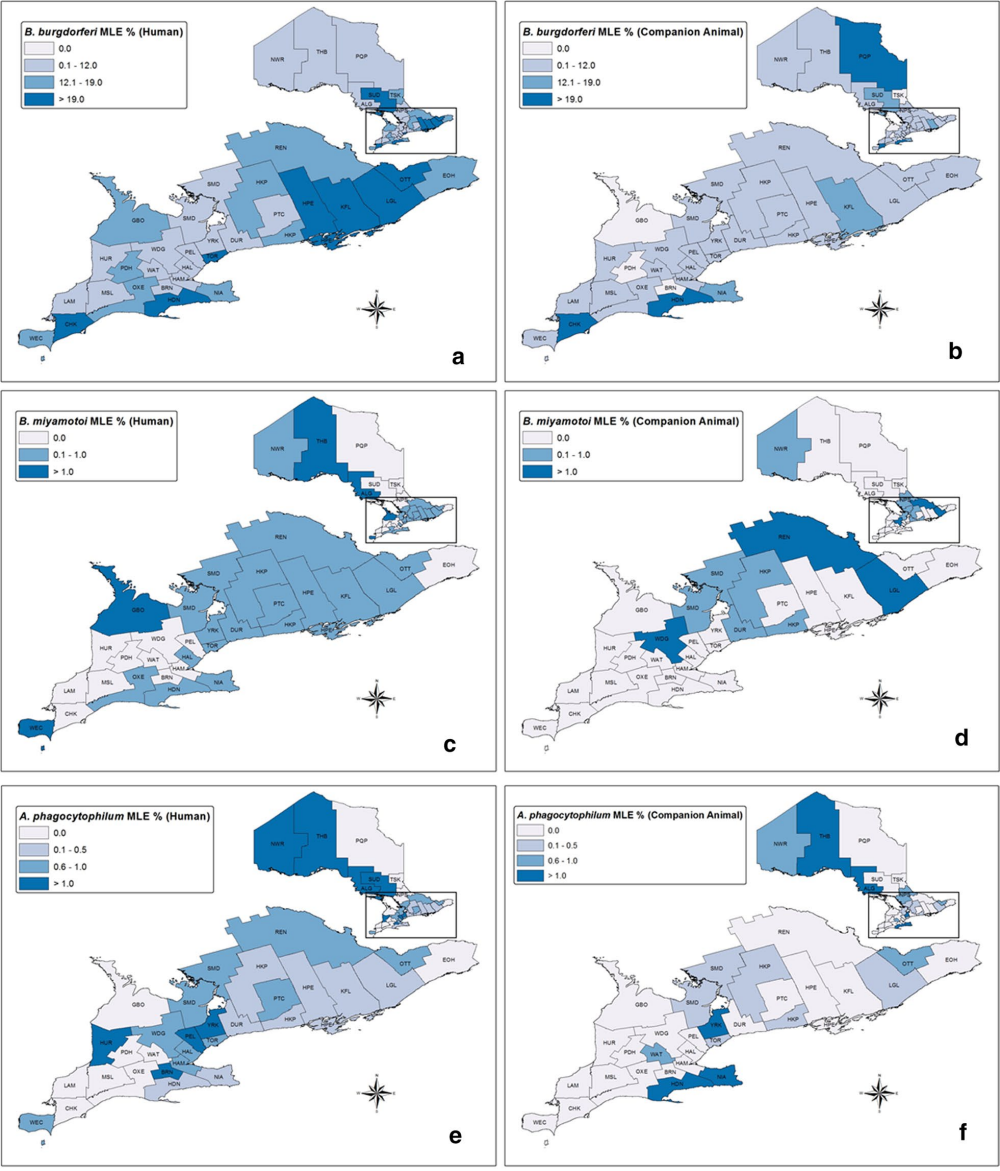

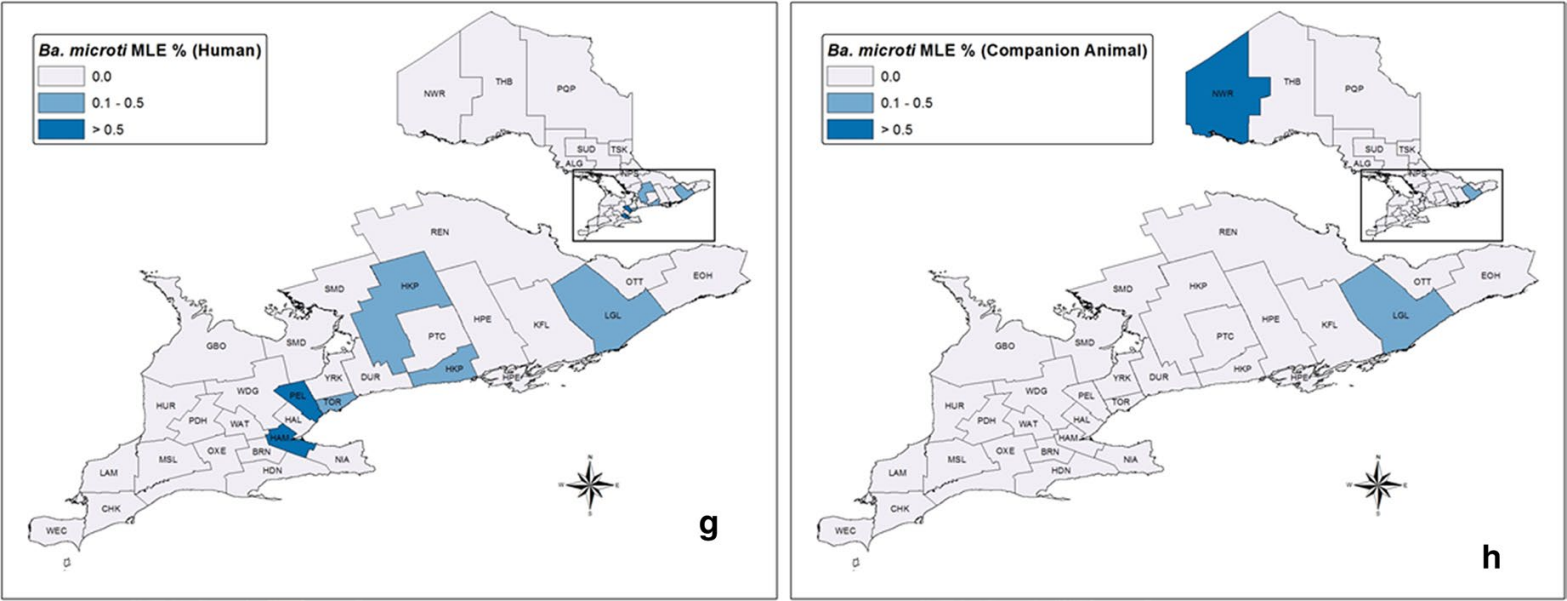
Pathogen prevalence (maximum likelihood estimate, MLE) in blacklegged ticks from humans (left) and companion animals (right): Ontario, Canada (2011–2017), excluding co-infections. **a**, **b** *Borrelia burgdorferi*; **c**, **d** *Borrelia miyamotoi*; **e**, **f** *Anaplasma phagocytophilum*; **g**, **h** *Babesia microti*.^*,**^. ^*^ALG Algoma District, *BRN* Brant County, *CHK* Chatham-Kent, *DUR* Durham Regional, *EOH* Eastern Ontario, *GBO* Grey Bruce, *HAL* Halton Regional, *HAM* City of Hamilton, *HDN* Haldimand-Norfolk, *HKP* Haliburton–Kawartha–Pine Ridge District, *HPE* Hastings and Prince Edward Counties, *HUR* Huron County, *KFL* Kingston-Frontenac and Lennox & Addington, *LAM* Lambton, *LGL* Leeds-Grenville and Lanark District, *MSL* Middlesex-London, *NIA* Niagara Regional, *NPS* North Bay Parry Sound District, *NWR* Northwestern, *OTT* City of Ottawa, *OXE* Oxford Elgin-St. Thomas, *PDH* Perth District, *PEL* Peel Regional, *PQP* Porcupine, *PTC* Peterborough County-City, *REN* Renfrew County and District, *SMD* Simcoe Muskoka District, *SUD* Sudbury and District, *THB* Thunder Bay District, *TOR* City of Toronto, *TSK* Timiskaming, *WAT* Waterloo, *WDG* Wellington-Dufferin-Guelph, *WEC* Windsor-Essex County, *YRK* York Regional

**Fig. 5:**
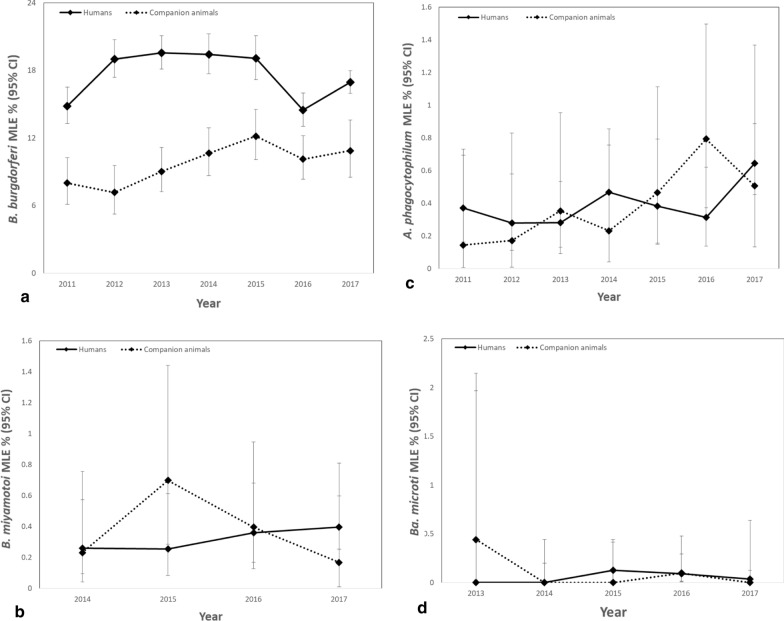
Pathogen prevalence (maximum likelihood estimate, MLE) in blacklegged ticks submitted from humans and companion animals: Ontario, Canada (2011–2017). **a** *Borrelia burgdorferi*, **b** *Borrelia miyamotoi*, **c** *Anaplasma phagocytophilum* and **d** *Babesia microti*

*Borrelia burgdorferi* prevalence (MLE) in blacklegged ticks from humans was 17.5% (95% CI 16.97–18.09%), with the highest prevalence in CHK, SUD, KFL, LGL, TOR, HDN, HPE and OTT (MLE > 19.0%) (Table 2, Additional file 1: Table S2, Fig. 4). *Borrelia miyamotoi* prevalence was 0.35% (95% CI 0.25–0.47%), with the highest prevalence in ALG, THB and GBO (MLE > 1.0%). *Anaplasma phagocytophilum* prevalence was 0.43% (95% CI 0.34–0.54%), with the highest prevalence in HUR, THB, ALG, SUD, PEL, NWR, BRN and YRK (MLE > 1.0%). *Babesia microti* prevalence was 0.054% (95% CI: 0.022–0.11%), with the highest prevalence in PEL and HAM (MLE ≥ 0.5%). Provincially, there was no significant increase or decrease in annual prevalence for any of the pathogens (Fig. 5).

**Table 2:** Pathogen prevalence in blacklegged ticks submitted from humans and companion animals: Ontario, Canada (2011–2017)

Pathogen	Humans	Companion animals	All hosts
	Infection prevalence, maximum likelihood estimate % (95% CI)		
B. burgdorferi*	17.5 (16.97–18.09)	9.9 (9.15–10.78)	15.8 (15.35–16.30)
B. miyamotoi**	0.35 (0.25–0.47)	0.39 (0.22–0.65)	0.36 (0.27–0.46)
A. phagocytophilum*	0.43 (0.34–0.54)	0.41 (0.26–0.60)	0.43 (0.35–0.52)
*Ba. microti*^†^	0.054 (0.022–0.11)	0.056 (0.010–0.18)	0.050 (0.025–0.10)

Co-infections^‡^	Infection prevalence, % (no. samples pos./no. samples tested)		
*B. burgdorferi* + B. miyamotoi**	0.11 (11/10,368)	0.16 (4/2479)	0.12 (15/12,847)
*B. burgdorferi* + A. phagocytophilum*	0.087 (9/10,368)	0.12 (3/2479)	0.093 (12/12,847)
*B. burgdorferi* + *Ba. microti*^†^	0.029 (3/10,368)	0.0 (0/2479)	0.023 (3/12,847)

*CI* confidence interval

*Blacklegged tick samples collected and tested from 2011 through 2017

**Blacklegged tick samples collected and tested from 2014 through 2017

^†^Blacklegged tick samples collected and tested from 2013 through 2017

^‡^Co-infection prevalence for single-tick samples only, by PHU (all hosts): *B. burgdorferi* + *B. miyamotoi*: HAL (*n* = 1), HDN (1), HPE (1), LGL (3), NIA (3), NPS (1), OTT (1), REN (1), SMD (2) and WEC (1); *B. burgdorferi* + *A. phagocytophilum*: ALG (1), DUR (1), HKP (2), HUR (1), KFL (2), LGL (2), NWR (1), OTT (3), REN (1), THB (2) and TOR (2) [multiple-tick samples: HKP (2), KFL (1), LGL (1), OTT (2)]; and *B. burgdorferi* + *Ba. microti*: HAM (1), HKP (2), LGL (1), NWR (1) and TOR (1) [multiple-tick samples: HKP (1), LGL (1), NWR (1)]

For co-infections, we analysed a subset of blacklegged ticks from humans limited to samples with a single tick (Table 2). There were 11 *B. burgdorferi* + *B. miyamotoi* (prevalence = 0.11%) co-infections in ticks from humans, followed by *B. burgdorferi* + *A*. *phagocytophilum* (*n* = 9, 0.068%) and *B. burgdorferi* + *Ba. microti* (*n* = 3, 0.029%). Co-infections occurred in 2011 (*n* = 1), 2012 (*n* = 1), 2014 (*n* = 2), 2015 (*n* = 5), 2016 (*n* = 4) and 2017 (*n* = 10).

### Blacklegged tick submissions from companion animals

We tested 4375 blacklegged tick samples submitted from companion animals for pathogens of interest. The majority of ticks were adult females (96.0%) and engorged (92.0%) (Table 1). Tick samples were primarily from dogs (92.6%). Total tick samples submitted varied from 447 to 812 per year (mean = 625 ± 56.5), with no increase in submissions during the surveillance period (Fig. 1). Most tick samples were submitted from April to June (36.7%) and October to November (52.7%) (Fig. 2). The highest rates of tick submissions were from HKP, LGL, THB, HPE, NPS, NWR, OTT, EOH and REN (> 75/100,000) (Additional file 1: Table S1, Fig. 3).

*Borrelia burgdorferi* prevalence (MLE) in blacklegged ticks from companion animals was 9.9% (95% CI: 9.15–10.78%), with the highest prevalence in PQP, CHK and HDN (MLE > 19.0%) (Table 2, Additional file 1: Table S2, Fig. 4). *Borrelia miyamotoi* prevalence was 0.39% (95% CI 0.22–0.65%), with the highest prevalence in WDG, LGL and REN (MLE > 1.0%). *Anaplasma phagocytophilum* prevalence was 0.41% (95% CI 0.26–0.60%), with highest prevalence in HDN, ALG, YRK, THB and NIA (MLE > 1.0%). *Babesia microti* prevalence was 0.056% (95% CI 0.010–0.18%), with highest prevalence in NWR (MLE > 0.7%). Provincially, there was no significant increase or decrease in annual prevalence for any of the pathogens (Fig. 5).

For co-infections, we analysed a subset of blacklegged ticks from companion animals limited to samples with a single tick (Table 2). There were four *B. burgdorferi* + *B. miyamotoi* (prevalence = 0.16%) co-infections in blacklegged ticks from companion animals, followed by *B. burgdorferi* + *A. phagocytophilum* (*n* = 3, 0.12%) co-infections. Co-infections occurred in 2015 (*n* = 2), 2016 (*n* = 4) and 2017 (*n* = 1).

### Comparing blacklegged tick submissions from humans and companion animals

The percent of blacklegged ticks submitted in the fall (October–November) was higher from companion animals (52.7%) than humans (45.8%) (*χ*^2^ = 77.7, *P* < 0.0001) (Fig. 2). The mean number of ticks submitted per sample was higher from companion animals (1.2 ± 0.022) than from humans (1.0 ± 0.0021) (*t* = 9.5; d*f* = 4457; *P* < 0.0001) (Table 1).

*Borrelia burgdorferi* prevalence was higher in ticks from humans (17.5%, 95% CI 16.97–18.09%) than from companion animals (9.9%, 95% CI 9.15–10.78%) (Table 2). There was no difference in pathogen prevalence, by host source, for the remaining pathogens.

**Fig. 6:**
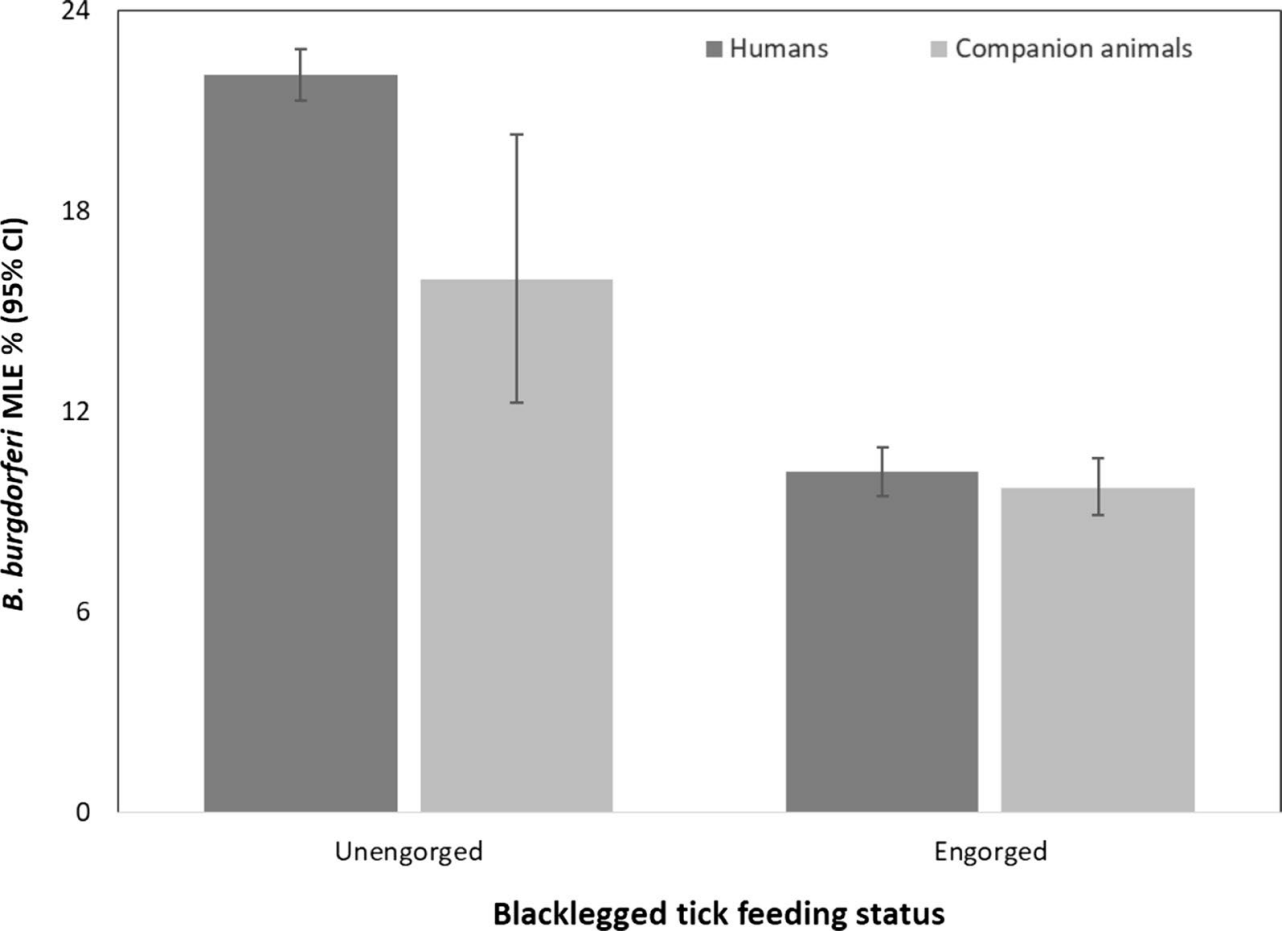
*Borrelia burgdorferi* prevalence (maximum likelihood estimate, MLE) by engorgement status in blacklegged ticks submitted from humans and companion animals: Ontario, Canada (2011–2017)

Percent engorgement was higher in ticks from companion animals (91.8%) than ticks from humans (37.8%) (*χ*^2^ = 3999; *P* < 0.0001) (Table 1). *Borrelia burgdorferi* prevalence was higher in unengorged ticks than engorged ticks from humans (22.1%, 95% CI 21.28–22.84% versus 10.2%, 95% CI 9.48–10.93%, respectively) and companion animals (16.0%, 95% CI 12.27–20.29% versus 9.7%, 95% CI 8.89–10.60%, respectively) (Fig. 6). Irrespective of host source, *B. burgdorferi* prevalence was higher in unengorged ticks (21.9%, 95% CI 21.12–22.65%) than engorged ticks (10.0%, 95% CI 9.45–10.56%). Engorgement status did not differ by host type for *A. phagocytophilum*, *B. miyamotoi* or *Ba. microti* (data not shown).

Co-infection prevalence in ticks from humans and companion animals did not differ for each co-infection pair: *B. burgdorferi* + *B. miyamotoi* (*χ*^2^ = 0.52, *P* = 0.50), *B. burgdorferi* + *A. phagocytophilum* (*χ*^2^ = 0.25, *P* = 0.62) and *B. burgdorferi* + *Ba. microti* (not applicable) (Table 2).

### Spatiotemporal relationship between the rates of submission from humans and animals over time

There were no consistent patterns evident across the PHUs between the annual rates of submissions from humans and companion animals (Additional file 1: Figure S1). Overdispersion was evident for all univariable Poisson models, so univariable negative binomial models were built. The incidence rate ratio (IRR) for the total number of ticks submitted from humans in each PHU (2011–2014) was significantly associated with the total number of ticks submitted from companion animals in each PHU for a subset of years (Additional file 1: Table S3). No other significant associations were detected.

## Discussion

During the study, there were over 21,500 blacklegged tick submissions from humans and companion animals. Blacklegged tick submission rates from humans were highest in the Eastern (EOH, HPE, KFL, LGL, REN), Central West (HDN), Central East (HKP, PTC), South West (LAM) and North West (NWR) regions of the province. The highest submission rates from companion animals were in the Eastern (EOH, HPE, LGL, OTT, REN), Central East (HKP), North East (NPS) and North West (NWR, THB) regions. We did not expect the relatively higher submission rates in certain PHUs (NPS, PTC, REN, THB), as these PHUs do not have known Lyme disease risk areas; however, these PHUs possibly represent areas where blacklegged ticks are emerging and/or are adjacent to PHUs with expanding tick populations. Corroborating our findings, public health officials declared new Lyme disease risk areas in REN and THB following the surveillance period for this analysis [36]. Increased blacklegged tick submission rates from companion animals in areas previously assumed not to have blacklegged tick populations are potentially an indicator of emerging local tick populations.

There was no temporal association in submission rates of blacklegged ticks from companion animals and submission rates from humans, indicating blacklegged tick submission rates from animals may not be a predictor of human tick exposure. Although several associations between blacklegged tick submission rates from humans and companion animals by PHU and year were significant based on univariable negative binomial regression from 2011 to 2014, the IRR hovered just over 1, indicating that the rates were approximately the same. This could be of interest if, for example, the rate of submissions from one host had an IRR ≥ 1 offset by a specific number of years, as this would provide preliminary evidence of a predictive relationship of animals for humans or vice versa. However, the significant associations did not appear to have a consistent pattern, and there were limited to no associations detected in later years. The literature indicates that dogs are more sensitive indicators of Lyme disease activity in an area than humans are, since dogs presumably have higher exposure to tick-infested habitat [37–39]. We did not examine Lyme disease rates in human and animal hosts; rather we examined blacklegged tick exposure. However, there is an opportunity to test this hypothesis in Ontario using canine and human serosurveys.

We acknowledge that the method of data collection differed based on host sources, resulting in more human submissions. PHUs actively requested and accepted human tick submissions, with the exception of EOH, KFL and LGL, which have not accepted ticks from passive surveillance since 2013. In contrast, PHUs did not accept submissions from companion animal hosts over this time; thus submissions only occurred if veterinarians were aware of the option to send the tick sample(s) directly to NML. Therefore, this study most likely does not reflect the true burden of ticks on companion animals, especially cats. Only 7% of submissions were from cats, and there is a growing body of evidence that cats may be exposed to more ticks than previously thought [40]. Given these limitations, it can be argued that if tick submissions from companion animals are relevant as sentinels for the risk of human tick bites, then a more systematic approach to collection of ticks from companion animals needs to be considered [11, 12, 37, 41]. Although reinstating the passive surveillance program for ticks from companion animals may not be possible given financial and human resource limitations, other models could be considered, such as collaboration with a network of veterinary clinics or submissions of digital images of ticks from the public (e.g., eTick, Pet Tick Tracker) [25, 42]. Continuing surveillance of ticks from human and animal hosts is an example of a One Health approach (including environmental and ecological factors), since the risk of tick bites and transmission of tick-borne pathogens can be assessed for both humans and companion animals, thus contributing to improved health of several species.

Overall, the most commonly detected pathogen was *B. burgdorferi* (prevalence = 16%), followed by considerably lower prevalence (< 1% each) for *B. miyamotoi*, *A. phagocytophilum* and *Ba. microti*. The low prevalence of several pathogens and co-infections was expected given the relatively recent emergence of blacklegged ticks in Ontario, compared to long-established populations (e.g., Wisconsin, USA) [43]. Pathogen prevalence in blacklegged ticks from human and companion animals did not change over the study period. Spatially, pathogen prevalence in blacklegged ticks was highest in PHUs with known blacklegged tick populations and relatively higher incidence of Lyme disease, which was expected [2, 20, 36]. The pathogens with relatively lower prevalence are not currently reportable to public health in Ontario; however, these pathogens will likely become more prevalent over time as blacklegged ticks continue to expand their range. There have been no reports in the literature of human infections in Ontario with *B. miyamotoi*. Locally acquired *A. phagocytophilum* infections are relatively rare in Ontario, with a human case in 2017 and an equine case in 2015 [44–46]. There are no reports of locally acquired human babesiosis cases in Ontario, with only travel- or blood transfusion-related human cases reported [47, 48]. Given that these pathogens are present in tick populations (although at low levels), consideration should be given to *B. miyamotoi*, *A. phagocytophilum* and *Ba. microti* as possible etiological agents where patients present with appropriate signs and symptoms.

Engorgement status of tick samples appears to have affected *B. burgdorferi* prevalence, where prevalence in engorged ticks, regardless of tick source, was less than half that of unengorged ticks. Interestingly, another study detected the opposite, where unfed blacklegged ticks had a lower prevalence of *B. burgdorferi* compared to engorged ones and ticks from people had a significantly lower infection prevalence than those from cats or dogs [49]. Dibernardo et al. [31] also noted a lower prevalence of *B. burgdorferi* in blacklegged ticks from people, but similar to our study, these authors found a lower infection prevalence when ticks were engorged compared to unengorged. Since *B. burgdorferi* tends to proliferate during the process of engorgement, this might predict that engorged ticks would have a higher likelihood of testing positive than unfed ticks [50]. Interestingly, in our study and other studies, *A. phagocytophilum* prevalence was similar, regardless of tick engorgement status and tick source [31]. Further controlled studies will provide a better understanding of the impacts of blood feeding status on the pathogen detection in ticks. We noted that ticks from companion animals were more frequently engorged than those submitted from humans. The disparity in engorgement status is likely the result of people noticing and removing ticks on themselves before they take a substantive blood meal, while ticks on companion animals go unnoticed and removed only once they have reached a large size and are noticed by their owners. The recovery of a higher percentage of nymphs and larvae from people compared to companion animals in Ontario further highlights the disparity in tick detection efficiency between the host types. With the possible impacts of engorgement status considered, we assume *B. burgdorferi* prevalence in blacklegged ticks is underestimated in our work. In future work, we plan to examine the role of blacklegged tick engorgement in pathogen prevalence, both spatially and temporally, in Ontario.

Our passive tick surveillance system likely underestimates the true risk of blacklegged tick exposure (and pathogen exposure) in the province. Not all people will notice a tick on themselves or their pets, and not everyone will submit a tick once detected. Non-submission of ticks is possibly due to variable tick awareness and healthcare-seeking behaviours of the public. We also likely underestimated pathogen prevalence in blacklegged ticks in PHUs that have stopped passive tick submissions (EOH, KFL, LGL); however, these PHUs are already characterized as high-risk areas and now concentrate their efforts on active surveillance.

## Conclusions

While we have observed *B. burgdorferi* in the province’s blacklegged ticks for several decades, surveillance and testing has uncovered additional tick-borne pathogens like *B. miyamotoi*, *A. phagocytophilum* and *Ba. microti* are also present at low prevalence both in ticks collected from humans and companion animals. Blacklegged tick and pathogen surveillance data from human and animal sources can be used as complementary data sources to monitor risk in human and companion animal populations and efforts are under consideration to unite surveillance efforts for the different host populations.

## Supplementary Information

**Additional file 1: Table S1.** Blacklegged tick sample submission rates by host and public health unit: Ontario, Canada (2011–2017). **Table S2.** Maximum likelihood estimate (MLE) prevalence of pathogens in blacklegged ticks from humans and companion animals, by public health unit: Ontario, Canada (2011–2017). **Table S3.** Univariable negative binomial regression to explore the influence of blacklegged tick submissions from animals on submissions from humans: Ontario, Canada (2011–2017). Bolded lines indicate significant associations (*P* < 0.05). **Figure S1.** Annual rates of blacklegged tick submissions per 100,000 population from humans and companion animals in each public health unit: Ontario, Canada (2011–2017).

## Data Availability

Information about PHO’s data request process is available on-line at https://www.publichealthontario.ca/en/About/Pages/data.aspx.

## Abbreviations

ALG: Algoma District
BRN: Brant County
CHK: Chatham-Kent
CI: Confidence interval
DUR: Durham Regional
EOH: Eastern Ontario
GBO: Grey Bruce
HAL: Halton Regional
HAM: City of Hamilton
HDN: Haldimand-Norfolk
HKP: Haliburton–Kawartha–Pine Ridge District
HPE: Hastings and Prince Edward Counties
HUR: Huron County
IRR: Incidence rate ratio
KFL: Kingston-Frontenac and Lennox & Addington
LAM: Lambton
LGL: Leeds-Grenville and Lanark District
MLE: Maximum likelihood estimate
MSL: Middlesex-London
NIA: Niagara Regional
NML: National Microbiology Laboratory
NPS: North Bay Parry Sound District
NWR: Northwestern
OTT: City of Ottawa
OXE: Oxford Elgin-St. Thomas
PDH: Perth District
PEL: Peel Regional
PHAC: Public Health Agency of Canada
PHU: Public health unit
PHO: Public Health Ontario
PQP: Porcupine
PTC: Peterborough County-City
REN: Renfrew County and District
SMD: Simcoe Muskoka District
SUD: Sudbury and District
THB: Thunder Bay District
TOR: City of Toronto
TSK: Timiskaming
USA: United States of America
WAT: Waterloo
WDG: Wellington-Dufferin-Guelph
WEC: Windsor-Essex County
YRK: York Regional
