# Supplementary material for: Monitoring the patterns of submission and presence of tick-borne pathogens in Ixodes scapularis collected from humans and companion animals in Ontario, Canada (2011–2017)
Source: Parasit Vectors. 2021 May 17;14:260. doi: 10.1186/s13071-021-04750-1 (PMC8127263; doi:10.1186/s13071-021-04750-1)

**Table S1** Blacklegged tick sample submission rates by host and public health unit: Ontario, Canada (2011–2017)

| Public health unit | Blacklegged tick submission rate per 100,000 population | |
| --- | --- | --- |
|  |  | |
|  | Human | Companion animal |
| ALG | 33.0 | 28.6 |
| BRN | 63.8 | 25.8 |
| CHK | 104.5 | 5.7 |
| DUR | 93.5 | 42.1 |
| EOH | 349.1 | 95.2 |
| GBO | 133.7 | 7.9 |
| HAL | 46.3 | 10.9 |
| HAM | 28.0 | 4.6 |
| HDN | 1,381.2 | 22.5 |
| HKP | 645.3 | 204.3 |
| HPE | 951.5 | 134.3 |
| HUR | 40.5 | 43.9 |
| KFL | 965.2 | 51.6 |
| LAM | 184.3 | 33.2 |
| LGL | 2,323.6 | 197.2 |
| MSL | 15.8 | 4.0 |
| NIA | 86.8 | 16.3 |
| NPS | 76.6 | 108.7 |
| NWR | 179.0 | 99.3 |
| OTT | 134.9 | 96.5 |
| OXE | 107.6 | 19.2 |
| PDH | 19.1 | 8.9 |
| PEL | 9.4 | 4.9 |
| PQP | 13.0 | 3.5 |
| PTC | 222.8 | 48.8 |
| REN | 319.4 | 77.0 |
| SMD | 62.3 | 43.4 |
| SUD | 31.1 | 45.7 |
| THB | 56.6 | 149.7 |
| TOR | 13.7 | 8.4 |
| TSK | 47.5 | 11.9 |
| WAT | 16.0 | 17.1 |
| WDG | 40.0 | 18.6 |
| WEC | 26.5 | 15.2 |
| YRK | 24.9 | 4.3 |
| Total (n) | 17,230 | 4,375 |
| All | 123 | 31.3 |

***Abbreviations*:** CA, companion animals; H, human; NA, not applicable (no tick samples submitted). ALG, Algoma District; BRN, Brant County; CHK, Chatham-Kent; DUR, Durham Regional; EOH, Eastern Ontario; GBO, Grey Bruce; HAL, Halton Regional; HAM, City of Hamilton; HDN, Haldimand-Norfolk; HKP, Haliburton-Kawartha-Pine Ridge District; HPE, Hastings and Prince Edward Counties; HUR, Huron County; KFL, Kingston-Frontenac and Lennox & Addington; LAM, Lambton; LGL, Leeds-Grenville and Lanark District; MSL, Middlesex-London; NIA, Niagara Regional; NPS, North Bay Parry Sound District; NWR, Northwestern; OTT, City of Ottawa; OXE, Oxford Elgin-St. Thomas; PDH, Perth District; PEL, Peel Regional; PQP, Porcupine; PTC, Peterborough County-City; REN, Renfrew County and District; SMD, Simcoe Muskoka District; SUD, Sudbury and District; THB, Thunder Bay District; TOR, City of Toronto; TSK, Timiskaming; WAT, Waterloo; WDG, Wellington-Dufferin-Guelph; WEC, Windsor-Essex County; and YRK, York Regional

^*^Blacklegged tick samples collected and tested from 2011 through 2017

^**^Blacklegged tick samples collected and tested from 2014 through 2017

^†^Blacklegged tick samples collected and tested from 2013 through 2017

**Table S2** Maximum likelihood estimate (MLE) prevalence of pathogens in blacklegged ticks from humans and companion animals, by public health unit: Ontario, Canada (2011–2017)

| Public health unit | Blacklegged ticks from humans | | | Blacklegged ticks from companion animals | | |
| --- | --- | --- | --- | --- | --- | --- |
|  | Pathogen prevalence (%) | Lower CI | Upper CI | Pathogen prevalence (%) | Lower CI | Upper CI |
| *Borrelia burgdorferi*^*^ | | |  |  |  |  |
| ALG | 10.53 | 3.51 | 23.30 | 11.76 | 3.95 | 25.75 |
| BRN | 8.38 | 3.99 | 15.26 | 0.00 | 0.00 | 8.52 |
| CHK | 28.57 | 20.82 | 37.39 | 46.48 | 14.01 | 82.98 |
| DUR | 9.78 | 7.68 | 12.24 | 8.06 | 5.45 | 11.45 |
| EOH | 17.73 | 15.08 | 20.64 | 11.73 | 8.09 | 16.32 |
| GBO | 13.96 | 9.87 | 18.98 | 0.00 | 0.00 | 21.24 |
| HAL | 8.52 | 5.62 | 12.31 | 9.60 | 4.02 | 18.87 |
| HAM | 8.69 | 5.05 | 13.80 | 3.56 | 0.21 | 15.95 |
| HDN | 20.37 | 18.43 | 22.41 | 20.77 | 8.92 | 38.72 |
| HKP | 14.90 | 12.97 | 17.00 | 6.89 | 4.78 | 9.57 |
| HPE | 19.72 | 17.83 | 21.73 | 11.74 | 8.27 | 16.07 |
| HUR | 8.33 | 1.52 | 24.55 | 7.12 | 1.30 | 21.21 |
| KFL | 21.34 | 19.59 | 23.17 | 13.99 | 9.31 | 20.16 |
| LAM | 5.00 | 2.81 | 8.19 | 1.60 | 0.09 | 7.43 |
| LGL | 21.31 | 20.07 | 22.60 | 10.52 | 8.20 | 13.24 |
| MSL | 8.00 | 3.34 | 15.83 | 9.98 | 1.84 | 28.74 |
| NIA | 15.62 | 12.32 | 19.40 | 16.83 | 9.96 | 26.04 |
| NPS | 10.00 | 5.22 | 17.05 | 11.58 | 7.46 | 16.98 |
| NWR | 9.93 | 5.91 | 15.47 | 9.30 | 4.94 | 15.98 |
| OTT | 19.30 | 17.26 | 21.47 | 10.93 | 9.19 | 12.88 |
| OXE | 13.81 | 9.73 | 18.85 | 9.28 | 3.10 | 20.69 |
| PDH | 13.33 | 2.47 | 36.93 | 0.00 | 0.00 | 35.43 |
| PEL | 11.43 | 6.93 | 17.50 | 4.05 | 1.08 | 10.54 |
| PQP | 9.09 | 0.53 | 36.34 | 57.45 | 12.17 | 97.19 |
| PTC | 7.04 | 4.63 | 10.22 | 9.08 | 4.10 | 17.09 |
| REN | 13.09 | 9.82 | 16.99 | 6.78 | 2.80 | 13.63 |
| SMD | 8.36 | 5.78 | 11.63 | 9.07 | 5.99 | 13.09 |
| SUD | 22.58 | 13.51 | 34.14 | 13.05 | 7.28 | 21.15 |
| THB | 6.82 | 2.84 | 13.58 | 9.01 | 5.83 | 13.21 |
| TOR | 20.83 | 17.08 | 25.01 | 10.93 | 7.67 | 15.05 |
| TSK | 12.50 | 2.31 | 34.98 | 0.00 | 0.00 | 48.99 |
| WAT | 10.11 | 5.08 | 17.69 | 7.03 | 3.15 | 13.37 |
| WDG | 8.55 | 4.45 | 14.67 | 7.27 | 2.41 | 16.49 |
| WEC | 15.87 | 10.01 | 23.48 | 11.12 | 5.02 | 20.79 |
| YRK | 8.49 | 5.69 | 12.11 | 8.25 | 3.12 | 17.43 |
| *Borrelia miyamotoi*^**^ | | |  |  |  |  |
| ALG | 3.70 | 0.21 | 16.55 | 0.00 | 0.00 | 15.36 |
| BRN | 0.00 | 0.00 | 4.23 | 0.00 | 0.00 | 8.91 |
| CHK | 0.00 | 0.00 | 4.18 | 0.00 | 0.00 | 37.17 |
| DUR | 0.49 | 0.13 | 1.31 | 0.96 | 0.17 | 3.08 |
| EOH | 0.00 | 0.00 | 1.21 | 0.00 | 0.00 | 2.89 |
| GBO | 1.30 | 0.23 | 4.18 | 0.00 | 0.00 | 29.11 |
| HAL | 0.39 | 0.02 | 1.87 | 0.00 | 0.00 | 8.02 |
| HAM | 0.00 | 0.00 | 2.55 | 0.00 | 0.00 | 14.77 |
| HDN | 0.35 | 0.11 | 0.83 | 0.00 | 0.00 | 16.61 |
| HKP | 0.10 | 0.01 | 0.48 | 0.73 | 0.13 | 2.37 |
| HPE | 0.32 | 0.10 | 0.77 | 0.00 | 0.00 | 1.89 |
| HUR | 0.00 | 0.00 | 14.87 | 0.00 | 0.00 | 12.70 |
| KFL | 0.21 | 0.01 | 1.02 | 0.00 | 0.00 | 3.29 |
| LAM | 0.00 | 0.00 | 1.52 | 0.00 | 0.00 | 5.89 |
| LGL | 0.56 | 0.23 | 1.15 | 1.74 | 0.57 | 4.10 |
| MSL | 0.00 | 0.00 | 5.27 | 0.00 | 0.00 | 18.25 |
| NIA | 0.83 | 0.22 | 2.22 | 0.00 | 0.00 | 17.59 |
| NPS | 0.00 | 0.00 | 5.58 | 0.60 | 0.03 | 2.88 |
| NWR | 0.81 | 0.05 | 3.86 | 0.75 | 0.04 | 3.55 |
| OTT | 0.18 | 0.03 | 0.59 | 0.00 | 0.00 | 0.60 |
| OXE | 0.47 | 0.03 | 2.25 | 0.00 | 0.00 | 10.04 |
| PDH | 0.00 | 0.00 | 21.53 | 0.00 | 0.00 | 39.03 |
| PEL | 0.00 | 0.00 | 2.93 | 0.00 | 0.00 | 7.69 |
| PQP | 0.00 | 0.00 | 25.88 | 0.00 | 0.00 | 49.91 |
| PTC | 0.33 | 0.02 | 1.57 | 0.00 | 0.00 | 6.79 |
| REN | 0.62 | 0.11 | 2.03 | 1.38 | 0.08 | 6.49 |
| SMD | 0.31 | 0.02 | 1.51 | 0.56 | 0.03 | 2.68 |
| SUD | 0.00 | 0.00 | 6.88 | 0.00 | 0.00 | 5.41 |
| THB | 1.79 | 0.10 | 8.31 | 0.00 | 0.00 | 2.48 |
| TOR | 0.27 | 0.02 | 1.29 | 0.00 | 0.00 | 2.55 |
| TSK | 0.00 | 0.00 | 22.81 | 0.00 | 0.00 | 56.15 |
| WAT | 0.00 | 0.00 | 4.69 | 0.00 | 0.00 | 4.62 |
| WDG | 0.00 | 0.00 | 3.59 | 2.08 | 0.12 | 9.63 |
| WEC | 1.03 | 0.06 | 4.87 | 0.00 | 0.00 | 9.18 |
| YRK | 0.36 | 0.02 | 1.73 | 0.00 | 0.00 | 6.71 |
| *Anaplasma phagocytophilum*^*^ | | |  |  |  |  |
| ALG | 2.63 | 0.15 | 12.03 | 2.94 | 0.17 | 13.34 |
| BRN | 1.04 | 0.06 | 4.92 | 0.00 | 0.00 | 8.52 |
| CHK | 0.00 | 0.00 | 3.31 | 0.00 | 0.00 | 34.06 |
| DUR | 0.46 | 0.12 | 1.22 | 0.00 | 0.00 | 1.14 |
| EOH | 0.00 | 0.00 | 0.52 | 0.00 | 0.00 | 1.51 |
| GBO | 0.00 | 0.00 | 1.70 | 0.00 | 0.00 | 21.24 |
| HAL | 0.74 | 0.13 | 2.39 | 0.00 | 0.00 | 5.74 |
| HAM | 0.62 | 0.04 | 2.96 | 0.00 | 0.00 | 11.97 |
| HDN | 0.38 | 0.15 | 0.78 | 3.20 | 0.19 | 14.38 |
| HKP | 0.50 | 0.20 | 1.02 | 0.22 | 0.01 | 1.08 |
| HPE | 0.31 | 0.11 | 0.68 | 0.00 | 0.00 | 1.35 |
| HUR | 4.17 | 0.24 | 18.43 | 0.00 | 0.00 | 11.92 |
| KFL | 0.44 | 0.22 | 0.81 | 0.00 | 0.00 | 1.45 |
| LAM | 0.00 | 0.00 | 1.44 | 0.00 | 0.00 | 5.64 |
| LGL | 0.24 | 0.12 | 0.43 | 0.18 | 0.01 | 0.89 |
| MSL | 0.00 | 0.00 | 4.87 | 0.00 | 0.00 | 15.99 |
| NIA | 0.49 | 0.09 | 1.60 | 1.16 | 0.07 | 5.46 |
| NPS | 0.00 | 0.00 | 3.70 | 0.56 | 0.03 | 2.69 |
| NWR | 1.33 | 0.24 | 4.30 | 0.70 | 0.04 | 3.30 |
| OTT | 0.52 | 0.23 | 1.02 | 0.70 | 0.33 | 1.32 |
| OXE | 0.00 | 0.00 | 1.61 | 0.00 | 0.00 | 8.14 |
| PDH | 0.00 | 0.00 | 20.39 | 0.00 | 0.00 | 35.43 |
| PEL | 1.43 | 0.26 | 4.59 | 0.00 | 0.00 | 4.93 |
| PQP | 0.00 | 0.00 | 25.88 | 0.00 | 0.00 | 45.07 |
| PTC | 0.61 | 0.11 | 1.97 | 0.00 | 0.00 | 4.66 |
| REN | 0.86 | 0.23 | 2.32 | 0.00 | 0.00 | 4.02 |
| SMD | 0.58 | 0.10 | 1.87 | 0.39 | 0.02 | 1.88 |
| SUD | 1.61 | 0.09 | 7.53 | 0.00 | 0.00 | 3.96 |
| THB | 3.41 | 0.90 | 8.92 | 1.29 | 0.34 | 3.44 |
| TOR | 0.73 | 0.19 | 1.97 | 0.33 | 0.02 | 1.58 |
| TSK | 0.00 | 0.00 | 19.36 | 0.00 | 0.00 | 48.99 |
| WAT | 0.00 | 0.00 | 4.14 | 1.00 | 0.06 | 4.73 |
| WDG | 0.85 | 0.05 | 4.06 | 0.00 | 0.00 | 6.52 |
| WEC | 0.87 | 0.05 | 4.16 | 0.00 | 0.00 | 5.65 |
| YRK | 1.01 | 0.27 | 2.71 | 1.59 | 0.09 | 7.47 |
| *Babesia microti*^†^ | | |  |  |  |  |
| ALG | 0.00 | 0.00 | 12.46 | 0.00 | 0.00 | 15.36 |
| BRN | 0.00 | 0.00 | 4.23 | 0.00 | 0.00 | 9.86 |
| CHK | 0.00 | 0.00 | 4.18 | 0.00 | 0.00 | 37.17 |
| DUR | 0.00 | 0.00 | 0.62 | 0.00 | 0.00 | 1.65 |
| EOH | 0.00 | 0.00 | 1.19 | 0.00 | 0.00 | 2.44 |
| GBO | 0.00 | 0.00 | 2.43 | 0.00 | 0.00 | 29.11 |
| HAL | 0.00 | 0.00 | 1.47 | 0.00 | 0.00 | 8.02 |
| HAM | 0.68 | 0.04 | 3.24 | 0.00 | 0.00 | 14.77 |
| HDN | 0.00 | 0.00 | 0.32 | 0.00 | 0.00 | 16.61 |
| HKP | 0.20 | 0.04 | 0.65 | 0.00 | 0.00 | 1.16 |
| HPE | 0.00 | 0.00 | 0.31 | 0.00 | 0.00 | 1.68 |
| HUR | 0.00 | 0.00 | 14.87 | 0.00 | 0.00 | 12.70 |
| KFL | 0.00 | 0.00 | 0.74 | 0.00 | 0.00 | 3.16 |
| LAM | 0.00 | 0.00 | 1.52 | 0.00 | 0.00 | 5.89 |
| LGL | 0.09 | 0.00 | 0.42 | 0.40 | 0.02 | 1.94 |
| MSL | 0.00 | 0.00 | 5.27 | 0.00 | 0.00 | 18.25 |
| NIA | 0.00 | 0.00 | 1.03 | 0.00 | 0.00 | 15.99 |
| NPS | 0.00 | 0.00 | 5.42 | 0.00 | 0.00 | 2.25 |
| NWR | 0.00 | 0.00 | 3.02 | 0.73 | 0.04 | 3.48 |
| OTT | 0.00 | 0.00 | 0.35 | 0.00 | 0.00 | 0.56 |
| OXE | 0.00 | 0.00 | 1.78 | 0.00 | 0.00 | 10.04 |
| PDH | 0.00 | 0.00 | 21.53 | 0.00 | 0.00 | 39.03 |
| PEL | 0.79 | 0.05 | 3.74 | 0.00 | 0.00 | 7.69 |
| PQP | 0.00 | 0.00 | 25.88 | 0.00 | 0.00 | 49.91 |
| PTC | 0.00 | 0.00 | 1.21 | 0.00 | 0.00 | 6.55 |
| REN | 0.00 | 0.00 | 1.19 | 0.00 | 0.00 | 4.89 |
| SMD | 0.00 | 0.00 | 1.19 | 0.00 | 0.00 | 2.09 |
| SUD | 0.00 | 0.00 | 6.88 | 0.00 | 0.00 | 5.41 |
| THB | 0.00 | 0.00 | 6.41 | 0.00 | 0.00 | 2.48 |
| TOR | 0.27 | 0.02 | 1.28 | 0.00 | 0.00 | 2.21 |
| TSK | 0.00 | 0.00 | 22.81 | 0.00 | 0.00 | 56.15 |
| WAT | 0.00 | 0.00 | 4.69 | 0.00 | 0.00 | 4.62 |
| WDG | 0.00 | 0.00 | 3.59 | 0.00 | 0.00 | 7.40 |
| WEC | 0.00 | 0.00 | 3.80 | 0.00 | 0.00 | 9.18 |
| YRK | 0.00 | 0.00 | 1.36 | 0.00 | 0.00 | 6.48 |

***Abbreviations*:** CA, companion animals; H, human; NA, not applicable (no tick samples submitted).

^*^Blacklegged tick samples collected and tested from 2011 through 2017

^**^Blacklegged tick collected and samples tested from 2014 through 2017

^†^Blacklegged tick samples collected and tested from 2013 through 2017

**Table S3** Univariable negative binomial regression to explore the influence of blacklegged tick submissions from animals on submissions from humans: Ontario, Canada (2011–2017). Bolded lines indicate significant associations (*P* < 0.05).

| Dependent variable: | Independent variable: | Incident rate ratio | *P*-value |
| --- | --- | --- | --- |
| No. ticks submitted from humans in each PHU by year | **No. ticks submitted from companion animals in each PHU by year** |  |  |
| 2011 | **2011** | **1.030** | **<0.001** |
| 2012 | **2012**  **2011** | **1.029**  **1.030** | **0.017**  **<0.001** |
| 2013 | 2013  **2012**  **2011** | 1.023  **1.027**  **1.028** | 0.058  **0.020**  **0.001** |
| 2014 | **2014**  **2013**  2012  **2011** | **1.030**  **1.021**  1.018  **1.017** | **0.022**  **0.039**  0.053  **0.036** |
| 2015 | **2015**  2014  2013  2012  2011 | **1.015**  1.015  1.011  1.008  1.008 | **0.046**  0.147  0.108  0.279  0.231 |
| 2016 | 2016  2015  2014  2013  2012  2011 | 1.005  1.009  1.010  1.007  1.003  1.002 | 0.337  0.167  0.222  0.159  0.628  0.670 |
| 2017 | 2017  2016  2015  2014  2013  2012  2011 | 1.009  1.006  1.010  1.010  1.009  1.003  1.002 | 0.328  0.298  0.114  0.194  0.110  0.534  0.658 |

**Figure S1** Annual rates of blacklegged tick submissions per 100,000 population from humans and companion animals in each public health unit: Ontario, Canada (2011–2017)


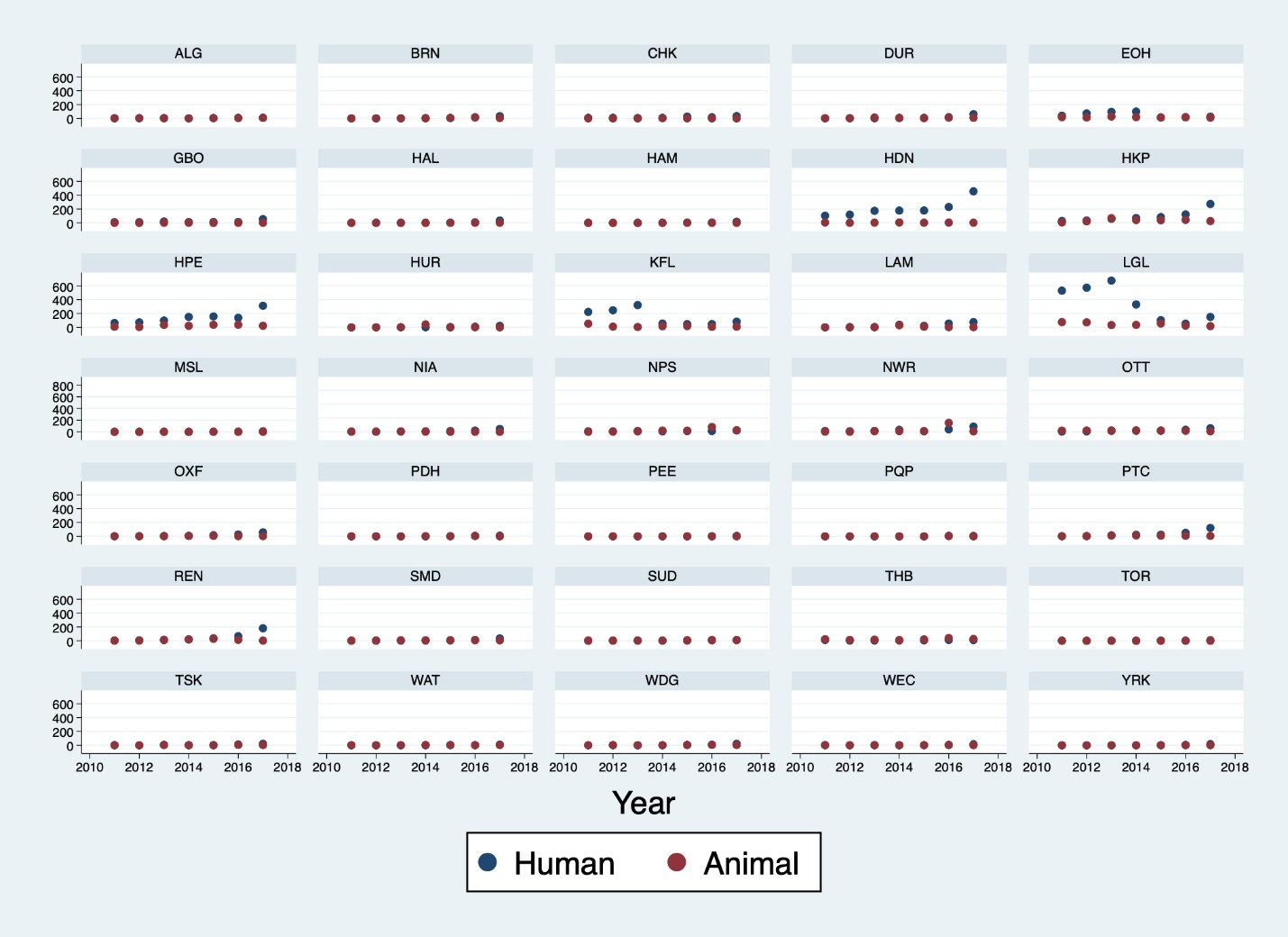

Supplement: Supplementary file 1 — Additional file 1: Table S1. Blacklegged tick sample submission rates by host and public health unit: Ontario, Canada (2011–2017). Table S2. Maximum likelihood estimate (MLE) prevalence of pathogens in blacklegged ticks from humans and companion animals, by public health unit: Ontario, Canada (2011–2017). Table S3. Univariable negative binomial regression to explore the influence of blacklegged tick submissions from animals on submissions from humans: Ontario, Canada (2011–2017). Bolded lines indicate significant associations (P < 0.05). Figure S1. Annual rates of blacklegged tick submissions per 100,000 population from humans and companion animals in each public health unit: Ontario, Canada (2011–2017). [file 13071_2021_4750_MOESM1_ESM.docx]
